# Are microRNAs suitable biomarkers of immunity to tuberculosis?

**DOI:** 10.1186/s40348-014-0008-9

**Published:** 2014-10-03

**Authors:** Bianca Ueberberg, Malte Kohns, Ertan Mayatepek, Marc Jacobsen

**Affiliations:** Department of General Pediatrics, Neonatology, and Pediatric Cardiology, University Children’s Hospital, Moorenstr. 5, 40225 Duesseldorf, Germany

**Keywords:** MicroRNAs, Tuberculosis, Biomarkers, Immunity

## Abstract

**Background:**

MicroRNAs (miRNAs) are crucial regulators of human immunity e.g. against *Mycobacterium tuberculosis*. Against the background of still alarming high mortality of tuberculosis effective biomarkers to improve diagnosis of *M. tuberculosis* infection and successful treatment are of major importance.

**Conclusions:**

This review summarizes recent surrogate tissue studies for identification of miRNA biomarker candidates in human tuberculosis with a special focus on reproducibility and conformance. In addition we provide assistance for the design of biomarker studies to circumvent major pitfalls.

## Basic requirements for miRNAs as biomarkers: impact on study designs

The crucial role of miRNAs in the regulation of immunity e.g. against infections is generally accepted. But do miRNAs also qualify as biomarkers in this context? Given the strict prerequisites of biomarkers that should reliably indicate (or predict) biological conditions [1], the applicability of miRNAs as biomarkers cannot be taken for granted. Importantly, the aim to identify biomarkers has also major implications on the study design. Especially, definition and characterization of study groups and samples in human studies are demanding and verification of biomarker candidates strongly depends on the comparability of different studies in this regard. Against this background, our mini-review aims to (i) summarize the current state of research on miRNAs as biomarkers in tuberculosis, (ii) evaluate study designs and comparability of biomarker studies, and (iii) point out minimal requirements and strategies to identify biomarkers.

## The need for biomarkers in tuberculosis

*Mycobacterium tuberculosis* (*M. tuberculosis*), the causative agent of human tuberculosis, is still a major threat to humankind. About 8 million new cases and more than 1.3 million deaths annually place tuberculosis among the top three fatal infections [2]. However, the vast majority of *M. tuberculosis*-infected individuals is capable of controlling the pathogen. These latently *M. tuberculosis*-infected (LTBI) individuals remain infected probably for lifetime. Diagnosis of tuberculosis and discrimination from LTBI in children are challenging because of the lack of clinical signs and imaging findings. Immunological tests (i.e. IFNγ release assays and tuberculin skin test) do also not discriminate active tuberculosis and LTBI. Since treatment regimen differs depending on the diagnosis, biomarkers for classification would be of great practical value. Protection against progression towards active disease is strongly dependent on an effective immune surveillance. Cellular immunity, especially CD4^+^ T cells and macrophages, are crucial players in this highly orchestrated host-pathogen interaction. The risk of adult LTBI to develop active tuberculosis is up to 10% per lifetime (with the majority of progression events occurring within the first two years; about 5%). Of note, infants and young children are highly susceptible to tuberculosis with untreated progression rates of up to 40% within the first year of infection. Biomarkers that reliably predict disease progression of LTBI would allow preventive treatment of highly susceptible individuals as it is done for young children. This way, the number of individuals that become infectious could be greatly reduced. As a consequence, also the number of ‘new’ LTBIs would decrease, a decisive step for eradication of tuberculosis.

A long treatment regimen about 6 to 9 months is necessary to cure tuberculosis patients. In addition, multidrug-resistant (MDR) *M. tuberculosis* strains complicate treatment courses. Especially insufficient adherence to chemotherapy against tuberculosis is a major problem in high incidence countries leading to treatment failure, development of MDR, as well as spread of *M. tuberculosis* infections. Shortened treatment e.g. by novel drugs or combination of existing medication would largely improve this situation and biomarkers that predict successful treatment could catalyze this process significantly. In childhood tuberculosis, prediction of effective treatment is particularly difficult since detection of *M. tuberculosis* in sputum fails in the majority of cases. Reduced pathogen load—defined by sputum conversion in adult tuberculosis patients and a hallmark for treatment efficacy—is therefore not definable in the majority of children. Moreover, bacterial load at treatment onset is expected to be lower in children than in adults. Biomarkers indicating bacterial load could contribute to the development of shorter treatment regimens.

Protective immunity against *M. tuberculosis* is predominantly based on the T-helper type 1 (T_H_1) mediated cellular arm of the host immune response and the fine-tuned interaction of T_H_1 cells with *M. tuberculosis*-infected macrophages. T-helper cell differentiation and plasticity was shown to be tightly regulated by miRNAs [3] and the same holds true for macrophages, the *M. tuberculosis* host cell population [4]. Therefore, the relevance of miRNAs for immune regulation in infectious diseases can be taken for granted.

## miRNAs in immunity against mycobacterial infections

The processes underlying the generation and regulation of miRNAs are reviewed in detail elsewhere [5]. In brief, miRNAs inhibit mRNA translation leading to mRNA target degradation and decreased protein expression. Several miRNA families regulate immune processes. miR-29, e.g., was shown to inhibit IFNγ expression of T cells [6]. miR-29 was able to block increased IFNγ levels, a typical feature of general miRNA knockout mice, and over-expression of miR-29 increased susceptibility to tuberculosis [6]. miR-21 was shown to be upregulated in macrophages after contact with *Mycobacterium bovis* BCG [7]. Wu et al. demonstrated miR-21-mediated inhibition of interleukin-12 in macrophages and, additionally, found increased apoptosis in dendritic cells due to miR-21 inhibition of bcl-2 [7]. They conclude that mycobacteria induce expression of miR-21 leading to impaired classical macrophage activation and dependent T_H_1 immunity. The mechanisms how mycobacteria interfere with host miRNA expression remain elusive. miR-155 has been intensively studied with regard to its role in immunity [8]. Macrophages infected with *M. tuberculosis* show differential miR-155 expression [9,10] and Kumar et al. identified the *M. tuberculosis* early secreted antigenic factor 6 (ESAT-6) as a crucial factor in this process [10]. The role of miR-155 in the interaction between macrophages and mycobacteria may have different aspects: interference with mycobacterial dormancy and inflammatory mediators (i.e. IL-6 and cyclooxygenase-2) [10]. Recently, Wang et al. demonstrated that miR-155 is involved in autophagy, an essential process of mycobacterial killing in host macrophages [11]. Taken together, previous studies strongly suggest a role of miRNAs in host immunity against tuberculosis.

For application as biomarkers of course, miRNA candidates must be detectable in surrogate tissue and availability of samples has to be ensured especially in tuberculosis endemic countries. Application of surrogate tissues/cells/body fluids comprises inherent jeopardy (i.e. differential cellular composition of blood or serum) that may confound results [1,12]. Hence, single biomarker or biomarker signatures must be robust enough to define or predict e.g. disease stages, treatment efficacy, or susceptibility despite of natural variability. To estimate the informative values of miRNA as biomarkers, several studies performed comparative expression analysis mainly in peripheral blood and sputum samples. These studies comprised global approaches to determine multiple miRNAs or measurement of single miRNA candidates. Results of these studies are discussed in the next chapters.

## miRNAs analyzed in blood and derived cells during *M. tuberculosis* infection

Peripheral blood and derived cell populations are frequently used for biomarker discovery and e.g. quantification of T-cell subpopulation proportions already made its way into clinical routine diagnostics of HIV/AIDS. In tuberculosis, candidate- and array-based global approaches were applied for miRNA analyses of peripheral blood and derived cells. An overview of miRNA biomarker studies and resulting candidates is provided in Table 1. The majority of studies focused on comparisons between patients with active TB, LTBIs, as well as non-*M. tuberculosis*-infected (TSTneg) controls. Wang et al. determined expression profiles of 955 miRNAs (human and human-viral) of enriched peripheral blood mononuclear cells (PBMCs) from TB patients and contacts with or without latent *M. tuberculosis* infection. Classification analyses did not discriminate between study groups but this may be due to small study group sizes [13]. Of 451 detectable miRNAs, a cluster of 17 miRNAs showed significant differences between active TB and *M. tuberculosis*-infected contacts (Table 1) [13]. Spinelli et al. used a candidate gene approach to determine expression of six miRNAs in PBMCs of TB patients and TSTneg individuals [14]. This study detected miR-424 to be upregulated in TB patients from both studies whereas no difference for miR-223 was found [14]. Detailed analyses of miR-223 have been performed by Dorhoi et al. who detected lower expression of miR-223 in PBMCs of TB patients as compared to LTBI and higher expression as compared to TSTneg [15]. Comparisons of pulmonary tissue samples revealed increased miR-223 expression in the lung of TB patients as compared to healthy controls [15]. Contrary findings for peripheral blood may therefore indicate differential migration activity of miR-223-expressing cells to affected tissue sites at different disease stages. Therefore, although miR-223 likely plays an important role in host immunity against TB, it may not qualify as a biomarker in surrogate tissue.

**Table 1:** **Biomarker studies of human blood and enriched immune cell populations**

**Study**	**Study type**	**Study group sizes**	**Upregulated**	**Downregulated**	**Overlap of differentially expressed miRNAs**
Wang et al. 2011 [13]	miRNA, array (955 miRNAs)	6 TB patients, 6 LTBIs	6 between TB and LTBI miR-21^a^ miR-223 miR-302a miR-424 miR-451 miR-486-5p	miR-130b^a^	miR-424
Spinelli et al. 2013 [14]	Candidate approach (6 miRNAs)	24 TB patients, 20 TSTneg	miR-424	miR-146a	
Wang et al. 2011 [13]	miRNA, array (955 miRNAs)	6 TB patients, 3 TSTneg	4 miRNAs miR-144 miR-365 miR-133a miR-424	3 miRNAs miR-500 miR-661 miR-892b	miR-144
Liu et al. 2011 [16]	miRNA array	3 TB patients, 3 controls (not further defined)	28 miRNA nv miR-144^a^	2 miRNAs nv	
Kleinsteuber et al. 2013 [18]	Candidate approach (29 miRNAs) enriched blood T cells	7 TB patients, 6 LTBIs, 3 TSTneg	No	4 miRNAs miR-21 miR-26a miR-29a miR-142-3p	
Fu et al. 2013 [17]	miRNA array (≈1,223 miRNAs) enriched blood T cells pooled from 4 donors	4 TB patients, 4 LTBIs, 4 TSTneg	6 miRNAs^b^ miR-340-5p miR-451a miR-32-5p miR-27a-3p miR-29a miR-29b	4 miRNAs^b^ miR-136-5p miR-4292 miR-H8^a^ miR-1915-3p miR-4258	miR-451 (Wang et al. 2011)

*nv* not verified by rtPCR, *TB* tuberculosis, *LTBI* latently *M. tuberculosis*-infected, *TSTneg* tuberkulin skin test negative individuals, *miRNAs* microRNAs.

^a^Indicates a mature miRNA species found at low levels from the opposite arm of a pre-miRNA hairpin.

^b^Within the miRNA candidate gene group differentially expressed as compared to TSTneg.

miR-144* was mentioned as a candidate regulator or IFN-γ expression before. Liu et al. performed global miRNA analysis of PBMCs from TB patients and healthy controls (three individuals per study group) [16]. This study identified 30 differentially regulated miRNAs but decided to focus on increased miR-144 expression in TB patients [16]. However, contrary findings with regard to miR-144* expression have been published. Wang et al. also found increased miR-144* expression in TB patients (only in comparison to TSTneg) [13] whereas no differential miR-144 expression was found by others [14]. Since miR-144* was described as an important T-cell factor in TB, different results may be due to confounding effects of cellular heterogeneity in peripheral blood [12]. Consequently, we and others performed miRNA expression analysis in enriched CD4^+^ T cells [17,18]. Kleinsteuber et al. analyzed miR-144* expression in CD4^+^ T cells but since it was not detectable in a subgroup of donors, miR-144* was excluded from further analyses [18]. In addition, a global miRNA array-based approach detected decreased miR-144 expression of CD4^+^ T cells in TB patients as compared to LTBI but these results of pooled sample analyses were not verified by quantitative PCR [17]. The same study focused on another promising candidate, namely miR-29, that was increased in CD4^+^ T cells from TB patients (compared to LTBI and TSTneg) [17]. In contrast, Kleinsteuber et al. detected decreased miR-29a of CD4^+^ T cells from TB patients compared to LTBI (but not TSTneg) [18]. Taken together, as for miR-223, a role for miR-144 and miR-29 in host immunity against TB is likely but the applicability of miR-29 as a biomarker has not been proven.

So far, only one study has been performed determining miRNA expression profiles of children with TB and LTBI [18]. Kleinsteuber et al. analyzed differentially expressed candidates of CD4^+^ T cells from adult TB patients. This study confirmed significant downregulation of miR-26a, miR-29a, and miR-142-3p in peripheral blood of children with TB compared to children with LTBI. In addition, a tendency of increased miR-26a, miR-29a, and miR-142-3p expression after recovery was found [18]. Nevertheless, the study of Kleinsteuber et al. also demonstrated marked interindividual differences of miRNA candidate expression (up to 10^5^-fold). This finding generally questions the applicability of miRNA as robust biomarkers for discrimination. At least, one would have to apply miRNA expression pattern of several miRNA, but studies that have sufficient statistical power are not available.

In conclusion, several data- or hypothesis-driven studies have been performed to identify miRNAs as surrogate tissue biomarkers in peripheral blood but highly promising candidates have not been identified.

## miRNAs detection in serum or plasma during human *M. tuberculosis* infection

Various studies identified miRNAs in human plasma associated with defined structures (i.e. exosomes and microvesicles apoptotic bodies) that are not degraded by plasma enzymes. There is arising evidence that circulating miRNAs exert biological functions e.g. as part of intercellular communication, and may be used as biomarkers for human diseases [19]. The appeal of using plasma miRNAs in clinical applications is high, as separation and preservation of plasma or serum samples is clinical routine also in *M. tuberculosis* endemic countries. However, variations in preanalytical processing of samples and lack of established endogenous controls limit the comparability of results [21].

So far, five studies have been performed to identify plasma miRNAs that distinguish between patients with pulmonary TB, LTBI, and TSTneg as well as other infections or malignant diseases. An overview and comparison of the results concerning discrimination between pulmonary TB and healthy subjects is given in Table 2. Fu et al. screened pooled serum samples of patients with pulmonary TB and matched healthy controls for differential expression of 1,223 miRNAs [20]. They identified 92 differentially expressed miRNAs (59 upregulated and 33 downregulated in TB patients). Three of these candidates were validated by qPCR in individual samples, but none of these three could be confirmed in later studies. Two differentially expressed candidates, i.e. miR-29a and miR-93*, were also assessed in sputum of the same patients. Notably, increased miR-29a expression was detected in sputum of TB patients. The same group investigated miRNA expression patterns of sputum in a second cohort and confirmed differential expression of miR-29a [21].

**Table 2:** **Biomarker studies of human blood serum and plasma**

**Study**	**Study type**	**Study group sizes**	**Upregulated**	**Downregulated**	**Overlap of differentially expressed miRNAs**
Abd-El-Fattah et al. 2013 [23]	Custom array for unspecified number of miRNAs (single samples)	29 TB, 37 healthy controls (no definition)	miR-182 miR-197		miR-197
Qi et al. 2012 [22]	Array for 667 miRNAs (pooled for study groups)	30 TB, 65 healthy controls (negative chest X-ray and IGRA, free from clinical symptoms of infection)	miR-361-5p miR-889 miR-576-3p		
					miR-25 miR-590-5p miR-885-5p
Miotto et al. 2013 [24]	Array for 671 miRNAs (pools of 10 individuals)	154 pulmonary TB, 105 healthy controls (negative IGRA or TST, no risk-factors for LTBI, no clinically significant condition) over 2 cohorts	miR-148a miR-16 miR-192 miR-193a-5p miR-25 miR-365 miR-451 miR-532-5p miR-590-5p miR-660 miR-885-5p miR-223^a^ miR-30e	let-7e miR-146	
					miR-365
Fu et al. 2011 [20]	Array for 1,223 miRNAs (pooled for study groups)	75 TB, 52 healthy controls (defined as ‘free of active and latent TB’)	miR-93^a^ miR-29a	miR-3125	
					miR-483-5p miR-22
Zhang et al. 2013 [25]	Deep sequencing (20 individual samples for each group)	128 pulmonary TB, 108 healthy controls (no definition)	miR-378 miR-483-5p miR-22 miR-29c	miR-101 miR-320b	

*TB* tuberculosis, *LTBI* latently *M. tuberculosis*-infected, *TSTneg* tuberkulin skin test negative individuals, *miRNAs* microRNAs.

^a^Indicates a mature miRNA species found at low levels from the opposite arm of a pre-miRNA hairpin.

Qi et al. compared sera of TB patients to healthy controls and patients with other diseases [22]. Overall, 667 miRNAs were determined in serum pools of TB patients and healthy controls by microarray analysis. This study identified 97 differentially expressed miRNAs and selected a set of ten for verification by quantitative PCR. A set of three miRNAs, i.e. miR-361-5p, miR-889, and miR-576-3p, was identified that specifically indicated TB disease. Differential expression of these candidate miRNAs was not found by any other study. Abd-el-Fattah et al. performed microarray-based analysis and validated results by qPCR to identify miRNAs for discrimination between pulmonary TB, pneumonia, lung cancer, pleural transudate, and matched controls [23]. In this study, a combination of increased miR-182 and miR-197 expression was found to be specific for TB. Correspondingly, Qi et al. also detected over-expression of miR-197 in TB patients [22].

Miotto et al. recruited two patient cohorts: (i) children with TB, TB/HIV co-infection and controls in Tanzania and Uganda as well as (ii) adult patients with TB (pulmonary and extra-pulmonary), LTBI, or other pulmonary infections and healthy controls in Italy [24]. This study compared array-based expression patterns of 671 miRNAs using sample pools of ten patients and 18 sex-matched individuals from the different subgroups. A cluster of 15 miRNAs distinguished between pulmonary TB and healthy controls. Within this set of markers, miR-192 was the only candidate significantly differentially expressed between the adult and the pediatric study groups. Comparing these results to the study of Qi et al., three miRNAs (miR-25, miR-590-5p, miR-885-5p) were found concordantly and let-7e disconcordantly regulated. Discrepancies may be due to different methods e.g. different endogenous controls used. Qi et al. used miR-16 as endogenous that has been found to be regulated by others [24].

Zhang et al. applied deep sequencing on serum samples between groups of patients with TB, pneumonia, chronic obstructive pulmonary disease, and lung cancer and healthy controls [25]. They identified a set of 15 differentially expressed miRNAs and a subset of six; namely, miR-378, miR-483-5p, miR-22, miR-29c, miR-101, and miR-320b classified the TB patients study group. miR-483-5p and miR-22 were also regulated concordantly in the study by Fu et al. whereas miR-101 was not different [20]. No differences for miR-29c were found in the study by Miotto et al. [24]. In conclusion, even some overlap of differentially expressed miRNAs between the studies existed; a common miRNA or miRNA pattern that classified TB patients was not found. miR-22, miR-25, miR-197, miR-365, miR-483-5p, miR-590-5p, and miR-885-5p are yet the most promising candidates since these miRNAs were validated for discrimination of TB and healthy controls in two studies (see Table 2).

## Conclusions of miRNA biomarker studies

Several studies characterized miRNAs of different surrogate tissues from *M. tuberculosis*-infected individuals and controls but common biomarker candidates have not been identified so far, neither in serum nor in blood cells. Several reasons may account for disconcordant results and possible confounding factors were (i) heterogeneous study designs including inconsistent cohort definitions and small study group sizes, (ii) marked interindividual variability of miRNA candidate expression especially in whole blood analyses, (iii) missing validation of targets from global analyses and different housekeeping miRNAs, (iv) inadequate statistical evaluation for candidate selection, as well as (v) absence of multifactorial classification approaches to define signatures of candidate miRNA biomarkers in most studies.

As a consequence, future biomarker studies should adhere to minimal prerequisites for conformity of study designs, case/control definitions, analytical settings, and data evaluation to ensure comparability of results. Table 3 summarizes some key points that should be considered for biomarker studies and highlights possible strategies and approaches to circumvent common pitfalls. Consideration of these points for the design and evaluation of biomarker studies will improve comparability of future studies and may lead to identification of suitable miRNA biomarkers.

**Table 3:** **Key points for the design and evaluation of miRNA biomarker studies**

**Topic**	**Major pitfall/mistake(s)**	**Strategies**
• Cohort definitions	• Insufficient inclusion criteria	• Exact definition of criteria for infection/disease
	• Donor/patient characteristics neglected	
		• Consideration of therapy/concomitant diseases
		• Focus on well defined study groups (e.g. children with tuberculosis/LTBI with a known index case)
• Small study group	• Insufficient statistical power due to multiple testing in ‘global’ miRNA analyses	• The definition of study group sizes markedly depends on (i) the number of miRNA candidates analyzed, (ii) the variability of target miRNA expression, (iii) the frequency of miRNA expressing target cells, and (iv) the desired sensitivity of the approach
	• High variability of miRNA candidate expression due to disease-independent regulatory mechanisms	
		• Include as many of the before mentioned parameters for study group size calculations
		• Cooperate with statisticians
• Tissue heterogeneity	• Differences in the proportions of miRNA expressing cellular subset confound analyses	• Usage of purified populations—as homogenous as possible
		• Characterization of heterogeneity (e.g. by flow cytometry) to deconfound results of heterogeneous tissues
• Statistical design and methods	• Application of inappropriate methods	• The definition of biomarkers requires discrimination
	• Significantly different is not the same as discrimination	• Discrimination tests (e.g. support vector machines and linear discriminance analysis) include training and test steps and study groups need to be defined accordingly
• Selection of miRNA targets	• Small study groups but extensive array analyses	• Focus on selected miRNAs targets for small study groups → hypothesis-driven approach
• ‘Housekeeping’ miRNAs	• No comparable internal standards	• Apply a group of ‘housekeeping’ miRNAs used in previous studies
